# Acculturation and use of traditional medicine among African migrant women in Sydney: a mixed method study

**DOI:** 10.1186/s12906-021-03424-w

**Published:** 2021-10-06

**Authors:** Zewdneh Shewamene, Tinashe Dune, Caroline A. Smith

**Affiliations:** 1 Ethiopian Health Insurance Agency (EHIA), P.O.Box 1176, Addis Ababa, Ethiopia; 2 NICM Health Research Institute, Western Sydney University, Penrith, NSW 2751 Australia; 3 School of Health Sciences, Translational Health Research Institute & Diabetes Obesity and Metabolism Translational Research Unit, Western Sydney University, Penrith, NSW 2751 Australia; 4 Graduate Research School, Western Sydney University, Penrith, NSW 2751 Australia

**Keywords:** Acculturation, Traditional medicine, African women, Migrants, Australia

## Abstract

**Background:**

More than 80% of the African population depend on traditional medicine as a primary healthcare. Although the African migrant community is increasing in Australia, there is no research documenting if and how African migrant communities have maintained or changed their use of traditional health practices after migration. This study aims to answer the following research questions: does acculturation influence the use of traditional medicine? and how are cultural health practices or beliefs manifested among African migrant women in Australia?

**Method:**

A mixed methods design which involved a cross-sectional survey (*n* = 319) and individual interviews (*n* = 15) was conducted. Survey data were analysed using SPSS (version 23) and logistic regression model was used to test associations. Qualitative data were analysed thematically using NVivo 11 software to identify themes and conceptual categories in the participants’ responses. The study was informed by acculturation theory.

**Result:**

Both the survey and the interview data indicated that cultural health practices were retained as an important form of healthcare for African migrant women in Sydney. The findings indicated that African migrants continued to use traditional medicines as part of their cultural identity and to build cohesive ethnic community to share traditional values and cultural practices. Women who relatively stayed for shorter period of time in Australia and migrated at a later age were more likely to use TM.

**Conclusion:**

Acculturation proxy measures increased the likelihood of TM use suggesting African migrant women retain their cultural health practices in Australia and use of TM was manifested as part of their cultural identity. The findings have implications to improve the provision of culturally sensitive and responsive health services when caring for African migrant women.

## Background

As defined by the World Health Organization [1], traditional medicine in this study refers to “the sum total of the knowledge, skills and practices based on the theories, beliefs and experiences indigenous to different cultures, whether explicable or not, used in the maintenance of health, as well as in the prevention, diagnosis, improvement or treatment of physical and mental illnesses”. Similarly, this study adopted the National Centre for Complementary and Integrative Medicine (NCCIM) definition for complementary and alternative medicine which states “group *of diverse medical and health care systems, practices, and products that are not generally considered part of conventional medicine”* [2].

Traditional medicine (TM) is an important component of the primary healthcare in Africa [3, 4]. Reports estimated that between 70 and 95% of the population in developing countries use TM [5, 6]. Greater than 80% of the African population use TM as a first line of health [3]. There are various suggested explanations for high utilisation of TMs. Scholars point to the conventional health care in Africa which has limitations of accessibility, availability and affordability to most of the population [7, 8]. In contrast, TM tends to be more affordable with both direct and indirect costs, easily accessible in villages, and individuals can self-apply them [9, 10].

Studies have suggested that when people migrate or arrive as refugee to a different country, they may take with them existing health beliefs and practices [11–13]. As such, in addition to using health care services offered in their new destination, migrants often practice their own traditional health remedies that they have brought with them [11, 14]. Currently, there is a lack of research that examines whether the use of TM in Africa also continues among migrant African communities in Western countries.

The extent to which such cultural health practices and beliefs are maintained, or changed, as people move from their native countries to new ones is important in addressing transcultural perspectives of migrants. In turn, this may help understand the needs of racial and ethnic minorities such as delivery of culturally appropriate health services, health seeking behaviors, compliance with treatment and attendance of health education programs [15].

Findings from a systematic review reported that women in Africa are extensively dependent on cultural health practices [16]. However, the experiences of these women after they resettle in Western countries, where biomedical care is predominant, remains largely unexplored. With the number of African ethnic minorities increasing in Australia [17, 18], this paper aims to answer the following research questions: 1) does acculturation affect the use of TM among African migrant women in Australia? and 2) how are their cultural health practices or beliefs manifested? in Australia? The study was informed by the acculturation theory to better understand how traditional health practices and beliefs change over time among African migrants.

## Methods

### Study design

This study used a mixed-method design which involved a cross-sectional survey and individual interviews. Combining quantitative and qualitative methods was important to strengthen the overall research design, utilizing the strengths of both approaches and generating more comprehensive and rich contextual data [19–21]. The criticism that qualitative research is subjective and provides few opportunities for generalisation across a large population can be overcome by quantitative methods that provides data on frequency of concepts or constructs from a representative sample [22–24].

The survey explored the influence of acculturation on use of TM considering women’s age at arrival, length of residency in Australia, and English language fluency. The interview examined in more detail women’s experiences and perceptions of using traditional after resettlement in Australia.

### Participants and sampling strategy

African migrant women residing across the Sydney metropolitan area who were born in Africa, aged ≥18 years, speak at least basic English Language (based on self-rated responses as ‘advanced’, ‘basic’, or ‘limited’), and had been a resident in Australia for at least 12 months were eligible to participate. Women who were in Australia on short term work or holiday visas were excluded.

Sample size determination for this survey was based on the need to ensure an adequate sample that permitted analysis of associations between specific predictor variables and use of traditional medicine. In a priori power analysis, the necessary sample size was computed as a function of user-specified values for the required significance level α, the desired statistical power 1-β, and the to-be detected population effect size [25]. A G*Power software program which is common in the social, behavioral, and biomedical sciences, was used to determine the required sample size in this study [25]. Accordingly, a total sample size of 299 subjects was required to detect moderate effects size of 0.20 [26] with 80% power and assuming 4 degrees of freedom when using Chi-square test of associations between key demographic or acculturation factors and use of traditional medicine.

When survey participants completed the questionnaires, they were invited to participate in follow up individual interviews. In addition to the inclusion criteria outlined under the survey, interview participants were selected purposively if they have previous experience of using traditional and complementary medicine. Fifteen eligible women were interviewed. After 13 interviews, no new themes emerged which indicated saturation was achieved.

Ethical approval was received from Western Sydney University Human Research Ethics Committee (ref: H1196). Informed consent was obtained from all women who participated in the study.

### Study promotion and recruitment procedures

The study was promoted through community consultations and networking with community associations, multicultural organisations, and business firms which were affiliated with the African communities in Sydney. Posters and flyers were displayed in prominent places including; shopping centers, train and bus stations, entertainment places, University and College campuses, restaurants, cafes, and African hair salons. For women expressing interest in the study, a link to the online questionnaire was sent through email, text messages and social media (Facebook, WhatsApp, and Viber). Optionally, paper-based questionnaires were hand delivered to individuals attending the organisations. A total of 332 women were sent the link to the online survey. A total of 302 paper-based questionnaires were distributed by hand. In total, 634 survey questionnaires were distributed using both the online and paper-based approaches. Of these, 348 (54.9%) surveys were returned (205 online and 143 paper-based). Of these, 29 questionnaires (25 online and 4 paper-based) were not fully completed, and therefore were disregarded, leaving a total number of 319 questionnaires to be analysed.

### Data collection and measures

Data was collected between October 2017 to January 2018. The survey explored socio-demographic characteristics including age, marital status, pregnancy and birth, income, education and employment status, religion, and country of origin were collected. The questionnaire was developed in English language and contained a total of 33 questions and completion time was approximately 15 min.

The survey also explored acculturation and patterns of TM use since migration to Australia. There were no standardised survey tools suitable to assess the use of TM and acculturation among African migrant communities in Western countries. Therefore, the present study developed questions based on findings from a comprehensive literature review [16, 27–31]. Proxy measures of acculturation including. Language, length of stay in Australia, and age at arrival were used in this study. Studies showed that proxy acculturation measures can be useful to assess acculturation in situations where use of a more comprehensive acculturation scale is not available [29, 31, 32]. A combination of 2-3 proxy measures have been indicated to effectively explore the effects of acculturation on health outcomes with greater flexibility [28, 30]. The most common proxy measures that have been shown to have high internal consistency were language spoken (during interview or at home), proportion of life lived in the host country, and generational status [30].

A pilot test of the survey was undertaken to examine face validity. The pilot was carried out among eight African-born women. Following the pilot study, several modifications were made including revising terms to improve the readability of the questionnaire.

Based on the survey findings, participants who had used TM were invited to participate in the interview. In total, 33 survey participants nominated their willingness to be interviewed when they completed the survey questionnaires. Of these, 15 eligible women were interviewed as a result of targeting more diversity in the sample to include different country of origin, length of stay in Australia and age. The development of the interview guide was informed by the findings from the survey, and the questioning in the interviews examined in more detail women’s experiences of using TM related to acculturation including the meaning of traditional medicine in their culture. Face-to-face and telephone interviews were conducted using a digital audio-recorder and notes were taken during and immediately following the interviews. The interviews were lasted between 40 and 80 min. All audio-recorded interviews were transcribed in full and results presented with pseudonyms.

### Data analysis

Survey data were checked for consistency, coded appropriately, and entered into SPSS software package version 23. Descriptive analysis using frequencies and proportions was conducted to describe the sample characteristics and distribution of variables. The relationship between TM use and acculturation factors was examined using binary logistic regression model. For all significance tests a *p*-value of 0.05 or less and a 95% confidence interval were used to quantify the associations between acculturation variables and use of TM. Bivariate logistic analysis was initially conducted with potential predictor variables to establish their association with use of TM. Following the bivariate analysis, multivariate logistic regression analysis was conducted to control for possible confounding variables and to compute the adjusted odds ratios (OR) for the explanatory variable. All variables with a *p*-value of less or equal to 0.25 were selected and entered simultaneously into a logistic regression model because the traditional level of 0.05 can fail to identify important variables [33]. A stepwise backward elimination method was employed to eventually produce the most parsimonious model. In backward elimination method, variables with greater *p*-value were sequentially eliminated until all variables in the model were *p* < 0.05 [34].

Qualitative data were analysed thematically using NVivo 11 software program [35] to identify themes, conceptual categories, commonalities, and differences in the participants’ responses. The analysis started by listening to the recoded audio and reading and re-reading the transcripts several times [36]. This process of familiarisation or immersion into the data helped the researcher to reflect on the entirety of the data and better understand and interpret the women’s views about TM. The next step was generating codes and using NVivo 11 to produce a concise matrix of key emerging ideas. Finally, a coding summary and report was produced with the sub-themes to answer the research questions. The first author conducted the data analysis and generated the summary codes. The second and third authors reviewed the interview transcripts, the coding summary and provided feedback for revisions to coding framework which were then incorporated into the qualitative data analysis by the first author. Pseudonyms have been used to protect the identity of women.

## Results

### Characteristics of participants

#### Survey participants

A total of 319 women came from 43 countries in Africa were participated in the survey. More than half of the study participants (171, 53.6%) arrived in Australia between the ages of 21 and 31 years. While 55 (20.7%) arrived in Australia after the age of 30 years, only 5 (1.6%) women migrated to Australia at the age of less than 10 years. Of the 319 participants, 111 (34.8%) were recent settlers (5 years or less) whereas 208 (65.2%) were long term settlers (greater than 5 years). One hundred twenty-four (38.9%) respondents migrated to Australia through the family or partner visa stream. Approximately one-third (100, 31.3%) of the participants resettled through a Humanitarian and refugee visa pathway. Currently, 130 (40.8%) women were citizens of Australia, while 123 (38.6%) were permanent residents (Table 1).

**Table 1:** Characteristics of survey participants

Characteristics		n (%)
Age on arrival	Less than 10 years	5 (1.6)
	11-20 years	77 (24.1)
	21-30 years	171 (53.6)
	Above 30 years	55 (20.7)
Time spent in Australia	1-5 years	111 (34.8)
	> 5 years	208 (65.2)
Visa pathway to come to Australia	family or partner	124 (38.9)
	Humanitarian or refugee	100 (31.3)
	Student	56 (17.6)
	Skilled/work	39 (12.2)
Current visa status	Citizen	130 (40.8)
	PR	123 (38.6)
	Temporary	60 (18.8)
	Refugee or asylum seeker	6 (1.9)
Self-rated English proficiency	Fluent	28 (8.8)
	Excellent	169 (53)
	Good or fair	122 (38.2)
Country of origin	Ethiopia	58 (18.2%)
	Ghana	13 (4.0%)
	Liberia	10 (3.1%)
	Nigeria	26 (8.2%)
	Sierra Leone	18 (5.6%)
	South Sudan	16 (5.0%)
	Sudan	51 (16.0%)
	Zimbabwe	18 (5.6%)
	Other 35 countries	109 (34.2%)

### Interview participants

Women in the interview also had diverse backgrounds from different regions in Africa, such as Nigeria, Cameroon, and Sierra Leone, in West Africa; Ethiopia, and Tanzania in East Africa; Rwanda and Gabon in Central Africa; Sudan in Northern Africa; and Zimbabwe in Southern Africa. The majority of participants had arrived to Australia on Family Visas (Table 2).

**Table 2:** Characteristics of interview participants

S. no	Name (age)	Age at arrival in Australia	Length of time in Australia, in year	Religion	Visa type on entry	English proficiency (Self rated)
1	Fiona (59)	38	21	Christian	Humanitarian /Refugee	Good
2	Zena (62)	30	32	Catholic	Humanitarian /Refugee	Excellent
3	Soy (46)	38	8	African traditional	Skilled/work	Good
4	Sim (33)	22	11	Christian	Family/partner	Good
5	Anna (42)	32	10	Christian	Humanitarian /Refugee	Excellent
6	Tia (28)	26	2	Christian	Student	Good
7	Mary (48)	18	30	Christian	Humanitarian /Refugee	Excellent
8	Eva (28)	26	2	Christian	Student	Fluent
9	Saly (48)	26	22	Christian	Family/partner	Excellent
10	Hiva (30)	19	11	Christian	Family/partner	Excellent
11	Ney (30)	10	20	Christian	Family/partner	Fluent
12	Mire (25)	23	2	Christian	Skilled/work	Good
13	Lita (35)	32	3	Christian	Family/partner	Excellent
14	Jay (26)	10	16	Christian	Student	Good
15	Ema (25)	20	5	Muslim	Family/partner	Good

### The influence of acculturation on use of traditional medicine

This section presents the integrated findings of the survey and interview data related to the influence of acculturation on use of TM among African migrant women. Generally, African migrant women retain the use of TM in Australia and they viewed it as part of their cultural identity.

Use of traditional or complementary medicines was high with 232 (72.7%) women using some form of traditional or complementary medicine (Table 3). The multivariate logistic regression analysis (Table 3) demonstrated that age on arrival, length of residency in Australia and English language proficiency were predictive acculturation factors of TM use among African migrant women in Australia. Women who migrated to Australia at the age of 20 or older were approximately 3.0 (95% CI, 1.54-5.33; *p* < 0.001) times more likely to use TM than those who arrived before the age of 20. Living in Australia for less than 5 years was significantly associated with an increased use of TM among African migrant women (OR, 3.1; 95% CI; 1.4-6.68; *p* = 0.004). Women with less English language fluency were 5.6 times more likely to use TM (95%CI; 2.58-12.11; *p* < 0.001).

**Table 3:** Multivariate logistic regression analysis for acculturation factors and use of traditional medicine

Variables		Total	Use of TM		^a^Adjusted OR	95%CI	*P*-value
			Yes	No			
Age on arrival	< 20 years	82	40 (48.8%)	42 (51.2%)	1	–	–
	> 21 years	235	192 (81.0%)	45 (19.0%)	2.9	1.54-5.33	< 0.001
Time spent in Australia	< 5 years	111	100 (90.1%)	11 (9.9%)	3.1	1.4-6.68	0.004
	> 5 years	208	132 (63.5%)	76 (36.5%)	1	–	–
Self-rated English proficiency	Good	122	112 (92%)	10 (8%)	5.6	2.58-12.11	< 0.001
	Excellent	169	101 (59.8%)	68 (40.2%)	1	–	–

^a^Adjusted ORs were calculated by controlling the refugee status of women (refugee or non-refugee) and women’s current migration status in Australia (citizens or non-citizens) which were not found significant during the bivariate analysis

The qualitative data analysis indicated that African migrant women retained their traditional health practices and beliefs despite greater access to conventional medical care in Australia. Some participants reported that they were using TM to a greater extent when they were new to Australia. For example, a participant who has been in Australia for the past 11 years commented:

*When I first came here [Australia] I had issues with delay [ed] medical attention mainly because I wouldn’t be comfortable, or I wouldn’t be able to express myself. I use [d] more traditional medicine instead. So yeah, it is something that changes over time may be because of the time I have been here, I sort of now seek medical help as quick as I can (Sim, 30 y/o).*

Another woman who has been in Australia for 32 years commented that during her first years of resettlement in Australia she preferred TM over conventional medical care because of her limited experience in navigating the health care system. Gradually she started to look to both health care systems even though she was unable to access some of her cultural traditional therapies that she knew in Africa.

*As time goes on, I am not too much into it [traditional medicine] as I was before in Africa or when I was new to Australia. Right after I came here I was just looking around many herbal clinics or the Chinese shops and asking people where to find these traditional doctors. But when I couldn’t find these things like in Africa, I actually prepare them by myself, at least some of them. But you know, gradually I am also depending on medications because I learn more about the health system in Australia which is very good and you don’t need to pay for it. So I am a kind of combining the both now. When I compare myself now and by the time I arrive to Australia long years ago, I am a bit depend [ent] on both medications and traditional medicine (Zena, 62 y/o).*

Respondents also reiterated that TM could be a means for social cohesiveness among family and friends to share traditional values and cultural practices.

*Many people try to bring their own traditional medicines and cultural foodstuffs when they go there [in Africa]. Some friends give me some medicines, like herbal ones, when they come from their village in Africa. They will give you something [families and friends], they share with you. If they bring some natural medicine from Africa, it is common for some people to share for someone else. Well it's not actually a lot who bring it to Australia, but still people do bring it here and you can find people who can share for you if you really need it most (Ema, 25y/o).*

In other cases, long term residents had a tendency to use both conventional and traditional health care. For example, Zena, whose been in Australia for 32 years stated, *“I still do believe in traditional medicines, but again I do believe again taking western medicines if needed.”*

For Sally the situation was different. Even though she has been in Australia for 22 years, she sometimes used TM that she learnt about from her parents. She also recalled how she had longed for the African version of TM available in Australia and the interaction with African traditional healers.

*I missed it [traditional medicine] a lot. I like the casualness, and the easy-going, and the connection with traditional healers. I still use some traditional medicine because I think it’s more also the connection that is who you are. So to be connected to your culture, and to you, what your mum was doing, what your dad was doing, it brings memory, and it does make you actually closer to home, to where you come from (Saly, 48 y/o).*

TM was deeply rooted and accepted in most African cultures. The interview participants pointed out how traditional health practices were important to them as a health choice and the need to continue these practices across generations.

*I will say to keep the originality of the culture. It's a part of our balance. So we need to keep it alive, because it has a kind of effectiveness. We used to live with it before, a long, long time ago. (Soy, 46 y/o).*

Participants also noted that in most African cultures, the knowledge and experience related to TM was orally transferred to the new generation. Lita said, *“so this traditional medicine has passed through a generation, so it’s like a generation passed and it has a healing power.”* Another participant also stated:

*Most of the recipes are well known with most of the elders in the family. It's more like when you have your own recipe that you use but it's something that originates from our culture. That has been tried and used for such purposes. This knowledge will be passed down to the new generation by oral means (Eva, 28 y/o).*

## Discussion

While specific acculturation proxy measures including older age at migration and short duration of residence increased the likelihood of TM use, African migrant women retain their cultural health practices in Australia. Overall, the use of TM was manifested as part of their cultural identity and cultural practices. Participants expressed their strong desire to maintain and pass along their cultural heritage about traditional medicine to their next generation.

We found that women who arrive in Australia after the age of 20 years are more likely to use traditional therapies than those who arrive at earlier age. This difference by age at arrival can potentially be explained by the limited exposure and adaptability of older migrants to the new lifestyles in Australia, as a result they may retain their cultural health practice and beliefs. This is in line with a previous study which documented that older migrants are less likely to acculturate or build new social networks and the presence of a network of people of the same ethnicity is likely to delay or inhibit acculturation [37]. Conversely, young migrants are more likely to be exposed to the lifestyles of their native-born counterparts and willing to acculturate [38].

In this study, shorter duration of residence in Australia was a significant predictive of TM use among African migrant women. This finding is consistent with a study among Asian-Americans that indicated those spent the majority of their life in US are less likely to use ethnic-specific complementary and alternative medicine [39]. This may be partly explained by the fact that women recently arrived in Australia may have a limited level of adaptation with the Western conventional medical care and, as a result, may stick to their traditional health practices and beliefs.

We also found that women who self-rated their English proficiency as low were more likely to use TM. This finding is in agreement with a study by Hsiano and colleagues that reported less English proficiency was related to greater ethnic-specific complementary and alternative medicine use among Asians and Latinos in US [40]. A possible explanation for our result may be the impact of language to navigate the conventional healthcare system in Australia [41] in which less English proficiency may limit women’s ability to effectively utilise available health services. This situation may lead to reliance on traditional alternative therapies that women can self-prescribe in most case [42].

The results from this study highlight the role cultural heritage plays in the use of TM among African migrant women. Interview findings found a strong desire to maintain and pass along their cultural heritage including TM. It seems possible that these results are due to the fact that TM has a significantly longer history than Western medicine in Africa and, as a result, most African culture is largely intertwined with it [16, 43]. In addition, women’s lack of previous experience in accessing modern health services in Africa may partly explain why they adhere to their cultural health practices and beliefs in Australia. Studies have shown that refugee and migrant women’s previous experiences in accessing health care influenced how they access the services in the new countries [41]. For example, Mengesha and colleagues found that refugee and migrant women’s participation in health promotion services were compromised by lack of previous experiences [41].

The impact of culture on health-related beliefs is complex and one’s attitudes and beliefs about health and disease are clearly shaped by the culture to which one belongs [44, 45]. Acculturation theory indicates that the stronger people identify with their traditional heritage, the greater the chance they will follow traditional health practices and beliefs derived from their cultures [46]. The use of culturally grounded traditional health practices and beliefs is related to a complex interaction of beliefs, values, perceptions, and religious and cultural orientations [46]. In general, this finding opposes the prediction that cultural health practices and beliefs will become a thing of the past when migrants move to a new destination country with a significant cultural variation. However, women use traditional medicine at a different level when specific migration factors are considered. For example, women who had stayed in Australia for shorter periods, moved to Australia at a later age, and have less English language proficiency were relatively high users of traditional medicine. This finding partly corroborates the preposition that groups and individuals who acculturate more slowly are more likely to rely on their cultural health practices [44]. As such, the use of acculturation theory in this study helped better explain the influence of acculturation on the use of traditional health practices of ethnic minorities such as African migrant women in Australia.

The implications of this study increase our understanding of how traditional health practices and beliefs are changed or maintained after African women resettled in Sydney. This is important to help develop culturally responsive approaches and community/population focused health partnerships with women from diverse ethnic, cultural, or language groups. Furthermore, these study findings will be of interest to policy makers and healthcare providers planning and delivering services for African migrant women.

### Limitation of the study

These findings must be considered in the context of the study’s limitations. Although multidimensional measures of acculturation provide a more comprehensive understanding of acculturation and its association with health outcomes, this study used proxy measures of acculturation. Since variables used to measure acculturation in one ethnic group may not work well in describing acculturation in other groups, use of proxy measures offer more flexibility than scale-based measures [31]. Studies also suggested that proxy measures are also effective in measuring different dimensions of acculturation [28, 31]. Another limitation of this study was that we did not explore the differences between African ethnic or cultural groups in terms of TM use and acculturation. We acknowledge that African migrant women in Australia represent diverse ethnic and cultural backgrounds. However, they still share some level of similarity within the Australia context, in terms of racialisation (despite their varying appearance), by geography (from Africa), culture (largely communal), and migration experience (having left Africa). Finally, generalisability of the study can be limited due to the fact that participants were required to speak at least basic English language and that may have led to a biased sample selection.

## Conclusion

TM is generally retained as an important form of health care by African migrant women in Sydney. Given that acculturation is a process in which African-born women’s health beliefs may change over time with an inclination towards the new culture in Australia, less acculturated women seem to rely more on TM. Findings from this study may have implications to better understand the transcultural perspectives of African migrants related to their cultural health practices and beliefs. In turn, this may enhance delivery of culturally appropriate health services and health education programs in Australia.

## Data Availability

The supporting materials used in this study are contained within the article.
